# Association between characteristics of swallowing-related muscles and trunk muscle mass

**DOI:** 10.1038/s41598-023-34905-2

**Published:** 2023-05-15

**Authors:** Kohei Yamaguchi, Kazuharu Nakagawa, Kanako Yoshimi, Chantaramanee Ariya, Ayako Nakane, Miki Ishii, Shohei Hasegawa, Haruka Tohara

**Affiliations:** 1 Department of Dysphagia Rehabilitation, Tokyo Medical and Dental University, 1-5-45 Yushima, Bunkyo-ku, Tokyo, 113-8510 Japan; 2 Department of Preventive Dentistry, Naresuan University, 99 Moo 9 Tambon Tha Pho, Mueang Phitsanulok, 65000 Thailand

**Keywords:** Health care, Signs and symptoms

## Abstract

Swallowing function is associated with systemic factors. Whether trunk or appendicular skeletal muscle mass is a better indicator of swallowing-related muscle characteristics in community-dwelling older adults is not clear. Hence, we investigated the association between the characteristics of swallowing-related muscles (e.g., mass and quality) and trunk muscle mass. Community-dwelling older adults aged ≥ 65 years (n = 141; men: n = 45, women: n = 96) were recruited for this cross-sectional observational study via a health survey conducted in 2018. Trunk muscle mass index (TMI) and appendicular skeletal muscle mass index (SMI) were measured using bioelectrical impedance analysis. Cross-sectional areas (CSAs) and echo intensity (EI) of the geniohyoid muscle (GHM) and tongue were evaluated using an ultrasonic diagnostic apparatus. Multiple regression analysis was used to examine the relationship of the characteristics of swallowing-related muscle with TMI and SMI. Multiple regression analysis showed that CSA of the GHM was positively associated with both TMI (B = 24.9, *p* < 0.001) and SMI (B = 13.7, *p* = 0.002). EIs of swallowing-related muscles were not associated with TMI and SMI. Trunk muscle mass was associated with swallowing-related muscle mass and not muscle quality. The results of this study shed light on the elucidation of association of dysphagia with TMI and SMI.

## Introduction

Deterioration of the swallowing function can be attributed to various factors, such as aging and weakening of swallowing-related muscles^1^. During swallowing, the tongue feeds the bolus to the pharynx, while the geniohyoid muscle (GHM) is primarily involved in laryngeal closure^2^. The cross-sectional areas (CSAs) of the GHM and tongue are associated with swallowing-related muscle strength^3,4^. GHM atrophy is associated with aspiration in community-dwelling older adults^5^. In a previous study that compared the characteristics of swallowing-related muscles of sarcopenic patients with and without dysphagia, those with dysphagia showed significantly decreased tongue muscle mass and quality^6^. The characteristics of swallowing-related muscles are indicators of swallowing function^7^. Ultrasonic diagnostic tools are useful for evaluating the characteristics of skeletal^8^ and swallowing-related muscles^4,9^.

The association between body composition, including appendicular skeletal muscle mass, and dysphagia has been demonstrated previously^10^. A prospective cohort study of 95 patients in convalescent hospitals reported that the loss of appendicular skeletal muscle mass was associated with dysphagia^11^. The role of skeletal muscles differs according to the site; the trunk muscles play an important role in postural maintenance^12^. The trunk muscles are associated with kyphosis severity^13^, wherein severe kyphosis leads to a forward head posture. The association between dysphagia and kyphosis has been reported^14^; the more severe the forward head posture, the longer the GHM, and the longer the GHM, the lower the jaw opening force^15^. Morphological features are also related to muscle strength in swallowing-related muscles^3^. A previous study showed that swallowing-related muscle strength, e.g., tongue pressure and jaw opening force, was associated with trunk muscle mass rather than appendicular skeletal muscle mass in community-dwelling older adults^12^.

However, whether trunk muscle mass is related to the characteristics of swallowing-related muscles (e.g., mass and quality) in community-dwelling older adults remains unclear. In light of the results of previous studies^12,16^, we hypothesized that trunk muscle mass is a better indicator of swallowing-related muscle characteristics than appendicular skeletal muscle mass in community-dwelling older adults. If the relationship between swallowing-related muscle characteristics and trunk muscle mass is clarified, the relationship between dysphagia and skeletal muscle mass can be elucidated. Therefore, this study aimed to clarify the relationship between trunk muscle mass and the characteristics of swallowing-related muscles.

## Materials and methods

### Participants

The participants of a health survey conducted in 2018 in Japan were recruited for this study. Older adults aged ≥ 65 years who could perform activities of daily living independently and were able to follow instructions were included. Those with a history of diseases affecting the muscles (e.g., neuromuscular disease and cerebrovascular disorder with paralysis), obvious dysphagia (e.g., symptoms of severe aspiration after surgery for head and neck tumors), a pacemaker, or missing relevant data were excluded. Some of the data analyzed in this study have been reported in previous studies^9,17^, except for the data related to Eating Assessment Tool-10 (EAT-10) and the Mini Nutritional Assessment-Short Form (MNA-SF). The sample size for this research was determined by calculating the detection power of multiple regression analysis using G power 3.1 (Kiel University, Kiel, Germany). In the post hoc test, α was 0.05, effect size was f^2^, and the number of participants was 141. All analyses showed a high detection power of ≥ 0.95. After providing sufficient written and oral explanation, written consent was obtained from each participant. This study was approved by the Ethics Committee of the Tokyo Medical and Dental University (ref: D2014-047), and the study protocol complied with the current ethical laws of Japan.

### Measurement of systemic factors

Body mass index was calculated by dividing body weight by height squared. Muscle mass was measured by direct segmental multifrequency bioelectrical impedance analysis, using In Body S10 (InBody Japan, Tokyo, Japan), at five measurement sites on the trunk and left and right limbs. The InBody S10 device measures impedance using a multifrequency alternating current of 1, 5, 50, 250, 500, and 1000 kHz, with eight electrodes attached to the left and right limbs. At the time of measurement, each participant sat on a chair in a relaxed state, with the back not hunched against the backrest, hands lowered away from the trunk, and knees not bent at right angles but extended slightly forward^12^. The trunk muscle mass index (TMI) was calculated by dividing the trunk muscle mass by square of the height^12^. The appendicular skeletal muscle mass index (SMI) was calculated by dividing the total muscle mass of the limbs by square of the height. Malnutrition was assessed using the MNA-SF^18^, which consists of six questions, with scores ranging as 0–2 or 0–3. A score of 0 indicates the worst condition; the higher the score, the better the condition. With a total of 14 points, 12–14, 8–11, and 0–7 points indicate good nutrition, malnutrition risk, and malnutrition, respectively.

### Measurement of oral and swallowing factors

Tooth loss was evaluated by experienced dentists or dental hygienists and classified according to the Eichner classification^19^. Ultrasonography of the GHM and tongue was performed using an ultrasonic diagnostic apparatus (Sonosite M-Turbo; Fujifilm, Tokyo, Japan). All measurements were performed by an experienced dentist. A 2–5-MHz convex probe with a depth of 9.2 cm was used. At the time of measurement, the probe was covered with a water-soluble transmission gel, and the participant was seated facing forward. The frequency and depth were constant for all measurements. The probe was placed at the line connecting the left and right second premolars, perpendicular to the Frankfurt plane^9^. Ultrasonographic images were analyzed using ImageJ software (version 1.49; National Institutes of Health, Bethesda, MD, USA). The CSA of the swallowing-related muscles was used as the proxy for swallowing-related muscle mass. Echo intensity (EI) was measured as an indicator of muscle quantity. The range of interest was set to include as much muscle as possible, without including the fascia. The average of two measurements was used as the measured value. Previous studies have shown sufficiently high intrarater reliability of ultrasonography for the GHM and tongue^9^. The EAT-10 is a self-reported questionnaire for dysphagia and consists of 10 questions, with points ranging from 0 to 4. Dysphagia is suspected when the total score is more than 3^20^.

### Statistical analysis

The normality of each item was confirmed using the Shapiro–Wilk test. *T*-test, Mann–Whitney test, and chi-square test were used for comparing the data between men and women. Further, participants were classified into low or high TMI and SMI groups by sex, using the 1st quartile of TMI and cut-off value determined by the Asian Working Group for Sarcopenia, respectively^21^. Coefficients of the correlations of the characteristics of swallowing-related muscles with TMI and SMI were calculated using the Pearson’s and Spearman’s correlation tests for parametric and non-parametric variables, respectively. To examine the relationship of the characteristics of swallowing-related muscles with TMI and SMI, multiple regression analysis was performed using swallowing-related muscle characteristics as dependent variables. The explanatory variables were age, sex, tooth loss, MNA-SF points, EAT-10 score, TMI, and SMI. The categorical variables were sex (0, men and 1, women) and tooth loss (1, Eichner A; 2, Eichner B; and 3, Eichner C). The forced input method was selected as the input method. To avoid multicollinearity, we checked whether the variance inflation factor was < 10.

## Results

**Figure 1:**
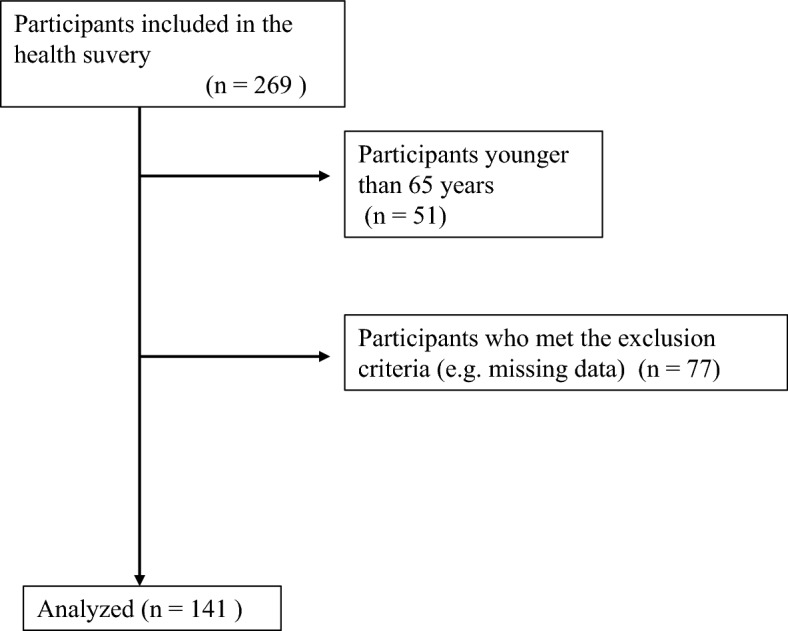
Flowchart of participants recruitment.

Initially, 269 participants were recruited, of which 51 were excluded because of age less than 65 years, and 77 were excluded according to the exclusion criteria (e.g. missing data) (Fig. 1). Finally, this study included 141 community-dwelling older adults aged 65–87 years (men: n = 46, women: n = 95). Sex differences were observed in the TMI, SMI, and characteristics of swallowing-related muscles (Table 1). The data were divided into two groups according low/high TMI and SMI, stratified by sex. For men, there were no significant differences in the low/high TMI, and the CSA of the tongue was significantly smaller in the low SMI group (*p* = 0.002) (Table 2). For women, the CSA of the GHM was significantly smaller in the low TMI group (*p* = 0.008) (Table 2). For both men and women, the CSA of the GHM was significantly positively correlated with the TMI and SMI (TMI: r = 0.50, *p* < 0.01 [men], r = 0.21, *p* < 0.05 [women]; SMI: r = 0.41, p < 0.01 [men], r = 0.21, *p* < 0.05 [women]), and the CSA of the tongue was significantly correlated with the SMI (r = 0.37, *p* < 0.05 [men], r = 0.24, *p* < 0.05 [women]) (Table 3).

**Table 1:** Participant characteristics.

	Total (N = 141)	Men (n = 46)	Women (n = 95)	Effect size	*p*-value
Age, years	71.6 ± 4.7	72.5 ± 5.2	71.1 ± 4.5	0.13	0.12^a^
BMI, kg/m^2^	23.1 ± 2.7	23.0 ± 3.0	23.1 ± 2.6	0.04	0.87^b^
TMI, kg/m^2^	7.2 ± 0.8	7.6 ± 0.8	6.9 ± 0.6	0.99	< 0.001^b^
SMI, kg/m^2^	6.6 ± 1.1	7.4 ± 1.2	6.3 ± 0.7	0.48	< 0.001^a^
CSA of GHM, mm^2^	164.4 ± 48.0	178.4 ± 57.6	157.7 ± 41.3	0.17	< 0.05^a^
CSA of tongue, mm^2^	1663.3 ± 275.9	1772.0 ± 267.8	1610.6 ± 265.6	0.61	0.001^b^
EI of GHM	18.5 ± 8.6	14.5 ± 6.1	20.5 ± 8.9	0.36	< 0.001^a^
EI of tongue	36.2 ± 7.9	32.3 ± 7.0	38.0 ± 7.7	0.77	< 0.001^b^
MNA-SF, points	12.0 ± 3.5	12.4 ± 2.9	11.9 ± 3.7	0.02	0.82^a^
EAT-10, scores	0.8 ± 1.8	1.0 ± 2.3	0.7 ± 1.5	0.05	0.57^a^
Tooth loss	Eichner A:81	Eichner A:30	Eichner A:51		0.43^c^
	Eichner B:48	Eichner B:13	Eichner B:35		
	Eichner C:12	Eichner C:3	Eichner C:9		

Values are presented as mean ± standard deviation.

^a^Mann–Whitney test.

^b^t-test.

^c^Chi-square test.

*BMI* body mass index, *SMI* skeletal muscle mass index, *TMI* trunk muscle mass index, *CSA* cross-sectional area, *EI* echo intensity, *GHM* geniohyoid muscle, *MNA-SF* mini nutritional assessment-short form, *EAT-10* eating assessment tool-10.

**Table 2:** Comparison of characteristics of swallowing-related muscles in groups formed according to the 1st quartile of trunk muscle mass and cut-off value of appendicular skeletal muscle mass.

	Men (n = 46)						Women (n = 95)					
	TMI			SMI			TMI			SMI		
	Low (n = 11)	High (n = 35)	*p*-value	Low (n = 10)	High (n = 36)	*p*-value	Low (n = 24)	High (n = 71)	*p*-value	Low (n = 20)	High (n = 75)	*p*-value
CSA of GHM	151.6 ± 37.1	186.9 ± 60.7	0.08^a^	159.5 ± 54.4	183.7 ± 58.1	0.25^a^	135.5 ± 32.1	165.2 ± 41.5	0.008^b^	141.6 ± 34.5	162.0 ± 42.0	0.12^b^
CSA of tongue	1718.9 ± 172.6	1788.7 ± 291.5	0.46^a^	1529.4 ± 247.0	1839.4 ± 234.6	0.001^a^	1547.1 ± 244.2	1632.1 ± 270.6	0.18^a^	1545.5 ± 227.5	1628.0 ± 273.6	0.22^a^
EI of GHM	15.8 ± 7.7	14.1 ± 5.5	0.42^a^	13.3 ± 5.5	14.8 ± 6.3	0.49^a^	21.0 ± 7.6	20.3 ± 9.4	0.66^b^	19.8 ± 8.5	20.7 ± 9.1	0.80^b^
EI of tongue	31.6 ± 9.7	32.5 ± 6.2	0.80^a^	31.5 ± 8.0	32.5 ± 6.9	0.70^a^	37.7 ± 8.5	38.1 ± 7.4	0.81^a^	35.4 ± 6.9	38.7 ± 7.8	0.09^a^

Values are presented as mean ± standard deviation.

*TMI* trunk muscle mass index, *SMI* skeletal muscle mass index, *GHM* geniohyoid muscle, *CSA* cross-sectional area, *EI* echo intensity.

^a^t-test.

^b^Mann–Whitney test.

**Table 3:** Correlation coefficients depicting the correlation of characteristics of swallowing-related muscles with TMI and SMI.

	Men		Women	
	TMI	SMI	TMI	SMI
CSA of GHM	0.50**^a^	0.41**^a^	0.21*^a^	0.21*^a^
CSA of tongue	0.14^b^	0.37*^a^	0.34**^b^	0.24*^a^
EI of GHM	− 0.12^b^	0.10^a^	− 0.05^a^	− 0.05^a^
EI of tongue	0.09^b^	− 0.10^a^	− 0.04^b^	0.12^a^

*CSA* cross-sectional area, *GHM* geniohyoid muscle, *EI* echo intensity, *TMI* trunk muscle mass index, *SMI* skeletal muscle mass index.

^a^Spearman’s correlation coefficient.

^b^Pearson’s correlation coefficient. *p < 0.05, **p < 0.01.

In the multiple regression analysis, the CSA of the GHM was positively associated with both TMI (B = 24.9, *p* < 0.001; Table 4) and SMI (B = 13.7, *p* = 0.002; Table 4). Multiple regression analysis with the CSA of the GHM as the dependent variable yielded standard partial regression coefficients of 0.40 and 0.30 for TMI and SMI, respectively. The CSA of the tongue was also associated with TMI (B = 105.2, *p* = 0.002) and SMI (B = 100.9, *p* < 0.001). The standard partial regression coefficients were 0.30 and 0.40 (Table 4) for TMI and SMI, respectively. The EIs of the swallowing-related muscles were not associated with TMI and SMI. All variance inflation factors were < 2.

**Table 4:** Multiple regression analysis with swallowing-related muscle characteristics as dependent variables.

Dependent variable	Independent variable	B (95% CI)	Standard partial regression coefficient	*p*-value	VIF	Adjusted R^2^
CSA of GHM	Age	− 2.37 (− 3.94 to − 0.80)	− 0.23	0.003	1.10	0.19
	Sex	− 4.90 (− 22.68 to 12.88)	− 0.06	0.51	1.32	
	MNA-SF point	− 0.72 (− 2.90 to 1.47)	− 0.05	0.52	1.09	
	Tooth loss	− 5.44 (− 16.76 to 5.88)	− 0.07	0.34	1.04	
	TMI	24.87 (13.93 to 35.80)	0. 40	< 0.001	1.34	
	EAT-10 score	1.44 (− 2.80 to 5.68)	0.05	0.50	1.08	
CSA of GHM	Age	− 2.07 (− 3.71 to − 0.42)	− 0.20	0.01	1.10	0.14
	Sex	− 6.57 (− 26.04 to 12.91)	− 0.06	0.51	1.51	
	MNA-SF point	− 0.27 (− 2.51 to 1.97)	− 0.02	0.81	1.07	
	Tooth loss	− 5.96 (− 17.71 to 5.78)	− 0.08	0.32	1.05	
	SMI	13.65 (5.13 to 22.17)	0.30	0.002	1.47	
	EAT-10 score	0.63 (− 3.73 to 4.98)	0.02	0.78	1.07	
CSA of tongue	Age	− 7.88 (− 17.24 to 1.48)	− 0.14	0. 10	1.06	0.13
	Sex	− 99.66 (− 205.88 to 6.56)	− 0.17	0.07	1.34	
	MNA-SF point	− 5.37 (− 18.40 to 7.66)	− 0.07	0.42	1.09	
	Tooth loss	30.64 (− 36.99 to 98.26)	0.07	0.37	1.04	
	TMI	105.19 (39.89 to 170.50)	0.29	0.002	1.34	
	EAT-10 score	7.80 (− 17.54 to 33.15)	0.05	0.54	1.08	
CSA of tongue	Age	− 5.12 (− 14.40 to 4.15)	− 0.09	0.34	1.51	0.17
	Sex	− 52.74 (− 162.50 to 57.03)	− 0.10	0.27	1.48	
	MNA-SF point	− 4.36 (− 16.98 to 8.26)	− 0.06	0.50	1.04	
	Tooth loss	23.38 (− 39.85 to 91.76)	0.06	0.50	1.04	
	SMI	100.93 (52.91 to 148.33)	0.39	< 0.001	1.47	
	EAT-10 score	7.78 (− 15.92 to 31.47)	0.05	0.52	1.04	
EI of GHM	Age	0.22 (− 0.07 to 0.51)	0.12	0.14	1.06	0.13
	Sex	5.15 (1.87 to 8.44)	0.28	0.002	1.34	
	MNA-SF point	0.42 (− 0.07 to 0.51)	0.17	0.25	1.09	
	Tooth loss	1.22 (− 0.87 to 3.31)	0.09	0.04	1.04	
	TMI	− 1.69 (− 3.70 to 0.33)	− 0.15	0.10	1.34	
	EAT-10 score	0.20 (− 0.58 to 0.99)	0.04	0.61	1.08	
EI of GHM	Age	0.23 (− 0.75 to − 0.16)	0.13	0.12	1.10	0.12
	Sex	6.61 (− 2.92 to 4.11)	0.36	< 0.001	1.51	
	MNA-SF point	0.37 (− 0.33 to 0.47)	0.15	0.08	1.07	
	Tooth loss	1.13 (− 0.87 to 3.36)	0.09	0.30	1.05	
	SMI	0.15 (− 0.65 to 2.42)	0.02	0.85	1.47	
	EAT-10 score	0.32 (− 1.46 to 0.11)	0.07	0.42	1.07	
EI of tongue	Age	0.26 (− 0.01 to 0.53)	0.16	0.05	1.34	0.13
	Sex	6.51 (3.47 to 9.56)	0.39	< 0.001	1.06	
	MNA-SF point	0.26 (− 0.11 to 0.63)	0.11	0.17	1.09	
	Tooth loss	0.76 (− 1.18 to 2.70)	0.06	0.44	1.04	
	TMI	0.25 (− 1.62 to 2.13)	0.02	0.79	1.34	
	EAT-10 score	0.56 (− 0.17 to 1.29)	0.13	0.13	1.08	
EI of tongue	Age	0.31 (0.04 to − 0.58)	0.19	0.03	1.10	0.15
	Sex	8.03 (4.85 to 11.21)	0.48	< 0.001	1.51	
	MNA-SF point	0.24 (− 0.33 to 0.47)	0.11	0.20	1.07	
	Tooth loss	0.61 (− 1.31 to 2.53)	0.05	0.53	1.05	
	SMI	1.37 (− 0.03 to 2.76)	0.18	0.06	1.47	
	EAT-10 score	0.63 (− 0.09 to 1.34)	0.14	0.09	1.07	

*B* partial regression coefficient, *R* coefficient of determination, *CI* confidence interval, *CSA* cross-sectional area, *GHM* geniohyoid muscle, *MNA-SF* mini nutritional assessment-short form, *SMI* skeletal muscle mass index, *TMI* trunk muscle mass index, *EAT-10* eating assessment tool-10, *EI* echo intensity, *VIF* variance inflation factor.

## Discussion

In this study, TMI and SMI were significantly and positively associated with the swallowing-related muscle mass. In contrast, they were not associated with the quality of swallowing-related muscles.

The GHM and tongue muscle mass are significantly associated with the jaw opening force and tongue pressure^3,22,23^. A cross-sectional study of 197 older adults demonstrated that the jaw opening force was associated with sarcopenia^24^, while another study involving 118 older adults demonstrated that the jaw opening force and tongue pressure were associated with TMI rather than SMI^12^. Tongue pressure is an independent factor associated with sarcopenia^25^. These previous studies support our finding of the association of the CSAs of the GHM and tongue with TMI and SMI.

The hyoid is a unique bone not connected to other bones; however, it is retained by soft tissues, such as muscles. Therefore, the hyoid bone position is easily affected by posture and head position. Anatomically, the GHM and tongue are connected to the hyoid bone; therefore, a forward head posture increases the GHM length^15^ and affects tongue position and morphology. When the mandibular bone is advanced by orthognathic surgery in patients with malocclusion, the length of the GHM increases significantly, and the CSA is greatly reduced compared to its preoperative state^26^. Tongue position and neurotransmission to the tongue affect postural retention^27,28^. Therefore, the CSAs of the GHM and tongue and trunk muscle mass involved in postural maintenance might have a significant association.

EI reflects the status of non-contractile tissue, such as intramuscular tissue^29^. In a previous report, EI of the quadriceps was reported to be more strongly associated with dysphagia than muscle mass in hospitalized older patients^30^. Muscle mass and EI were negatively associated in skeletal and swallowing-related muscles^7,9,31^. Muscle atrophy can promote adipose differentiation through the involvement of muscle satellite cells and mesenchymal progenitor cells^32,33^. In contrast, in this study, EI of swallowing-related muscles was not associated with TMI or SMI. It has also been reported that the properties of preadipocytes that differentiate into new adipocytes vary widely in a site-specific manner^34^. Although the relationships between trunk, limb, and swallowing-related muscles have been shown in terms of muscle mass, it is necessary to examine the relationship between intramuscular adipose tissue of each muscle.

This study had some limitations. Since this was an observational study, the causal relationship between swallowing-related muscle characteristics and trunk muscle mass could not be determined. Longitudinal studies with a larger sample size are necessary to demonstrate the causal relationship between swallowing-related muscle characteristics and strength, trunk muscle mass, and appendicular skeletal muscle mass. Furthermore, in this study, 13 participants were suspected of having dysphagia, with an EAT-10 score of ≥ 3, accounting for 9.2% of the total sample; this was considerably lower than the 15% prevalence of dysphagia in the community shown in a previous study^35^.

Despite these limitations, this study provided important insights for clinicians. Older adults with increased trunk and appendicular skeletal muscle mass have increased swallowing-related muscle mass. TMI and SMI are good indicators of swallowing-related muscle mass but not of swallowing-related muscle quality, regardless of the site. Swallowing-related muscles could be seamlessly related with trunk and appendicular skeletal muscles.

## Conclusions

We demonstrated that the CSAs of the GHM and tongue were associated with TMI and SMI, whereas TMI and SMI were not significantly associated with the quality of swallowing-related muscles. TMI and SMI are useful indicators of swallowing-related muscle mass. Insights on the relationship between the mass and quality of swallowing-related muscles and systemic factors can facilitate future studies on the pathology of dysphagia due to sarcopenia.

## Data Availability

The data that supports the findings of this study are available from the corresponding author, [KY], upon reasonable request.
